# Bioavailability study of Enoxaparin Sodium Chemi (80 mg/0.8 mL) and Clexane (80 mg/0.8 mL) subcutaneous injection in healthy adults 

**DOI:** 10.5414/CP204022

**Published:** 2021-08-17

**Authors:** Salvatore Febbraro, Javier Leal Martínez-Bujanda, Concepción Nieto Magro, Paolo Bettica

**Affiliations:** 1 Senior Medical Director, PRA Health Sciences, Reading, UK,; 2 ITF Research Pharma S.L.U, Alcobendas, Spain, and; 3 Italfarmaco SpA, Milan, Italy

**Keywords:** enoxaparin, subcutaneous, bioavailability, bioequivalence, anti-Xa activity

## Abstract

Objective: The present study compared the bioavailability of subcutaneous (s.c.) Chemi Enoxaparin with Clexane (80 mg/0.8 mL) under fasting conditions in healthy subjects. Materials and methods: This study was an open-label, randomized, single-dose, two-treatment period crossover study. We included healthy male and female subjects aged 18 – 55 years with a body mass index of 18 – 30 kg/m^2^. The primary pharmacodynamic endpoints were anti-FIIa and anti-FXa activity. Bioequivalence was achieved when the 95% confidence interval (CI) for the geometric means of C_max_ and AUC_0–t_ was between 80.00 and 125.00%. Results: 47 subjects were randomized for the treatment sequences. The 95% CI of the ratios of the geometric least squared means of anti-FXa activity was 96.28 – 102.65 IU/mL for C_max_ and 100.67 – 105.15 h×IU/mL for the AUC_0–t_ of Chemi Enoxaparin compared with those of Clexane, and for anti-FIIa activity, they were 86.65 – 96.73 IU/mL for the C_max_ and 87.72 – 97.25 h×IU/mL AUC_0–t_, which met the criterion for bioequivalence. The number of subjects reporting at least 1 treatment-emergent adverse event (TEAE) was low, mostly of mild severity, and similar for both compounds. Conclusion: Chemi enoxaparin is bioequivalent to the reference enoxaparin, and both compounds show similar tolerability and safety profiles.

Clinical trials registry: EUDRACT NUMBER: 2014-002690-11 

**What is known about this subject**

- Enoxaparin is effective in several arterial thromboembolic disorders, such as unstable angina, acute coronary syndrome, and ST-elevation myocardial infarction, in patients who undergo elective percutaneous coronary intervention, and in the prophylaxis or treatment of venous thromboembolism.
- The increasing incorporation of biological medicines, such as low-molecular-weight heparin, in the treatment of many diseases has significantly affected pharmaceutical budgets in multiple therapeutic areas and increased pharmaceutical expense.
- The European Commission stated that the availability of biosimilar medicinal products enhances competition, with the potential to improve patient access to biological medicines and contribute to the financial sustainability of European Union healthcare systems.

**What this study adds**

- This pivotal, crossover study compared the bioavailability of enoxaparin from the test investigational medicinal product (Chemi Enoxaparin) with the reference investigational medicinal product (Clexane, manufactured by Sanofi) when administered as an 80-mg/0.8-mL subcutaneous (s.c.) injection under fasting conditions in healthy subjects.
- This single-dose, pivotal study demonstrated that Chemi Enoxaparin (80 mg, s.c.) is bioequivalent to the branded Clexane (80 mg, s.c.), and both compounds show similar tolerability and safety profiles.

## Introduction 

Enoxaparin is a low-molecular-weight heparin (LMWH) that, like unfractionated heparin, acts at the final common pathway of the coagulation cascade. It binds to anti-thrombin III, and the complex of enoxaparin-anti-thrombin III inactivates clotting factors Xa, IIa, and IXa, which are responsible for the conversion of prothrombin to thrombin, which is responsible for the conversion of fibrinogen into fibrin and clot formation [1]. Enoxaparin has a higher ratio of anti-Xa to anti-IIa activity, which may be related with a lower trend of hemorrhagic effect [2]. 

A large number of randomized clinical trials have demonstrated that enoxaparin was effective in several arterial thromboembolic disorders, such as unstable angina, acute coronary syndrome, and ST-elevation myocardial infarction, in patients who underwent elective percutaneous coronary intervention, and in the prophylaxis or treatment of venous thromboembolism [3]. It was first approved and marketed in France in 1987, and thereafter became available in most countries worldwide, including the United States in 1993. It is currently indicated in the European Union (EU) for prophylaxis of venous thromboembolic disease in moderate- and high-risk surgical patients or medical patients with an acute illness, such as acute heart failure, respiratory insufficiency, severe infections or rheumatic diseases; in those with reduced mobility who are at increased risk of venous thromboembolism; for the treatment of deep vein thrombosis and pulmonary embolism (excluding embolisms that require thrombolytic therapy or surgery); for the prevention of thrombus formation in extra-corporeal circulation during hemodialysis; and for the treatment of acute coronary syndrome, including treatment of unstable angina and non-ST-segment elevation myocardial infarction, in combination with oral acetylsalicylic acid, and the treatment of acute ST-segment elevation myocardial infarction [4]. Compared with other parenteral anticoagulants, including unfractionated heparin, enoxaparin has several advantages: rapid and predictable absorption, higher bioavailability, and once- or twice-daily dosing, which favor home-based administration without the need of monitoring [3]. 

However, the increasing incorporation of biological medicines, such as LMWH, in the treatment of many diseases has significantly affected pharmaceutical budgets in multiple therapeutic areas and increased pharmaceutical expense [5]. The European Commission stated that the availability of biosimilar medicinal products enhances competition, with the potential to improve patient access to biological medicines and contribute to the financial sustainability of EU healthcare systems [6]. The savings in pharmaceutical costs with the use of biosimilars was estimated at 20 – 30% [7]. 

Chemi S.p.A., Milan, Italy, (a company of the Italfarmaco Group) developed a formulation of enoxaparin, enoxaparin sodium chemi (Chemi Enoxaparin), which was approved by the U.S. FDA in 2014 and has over 5 years of commercial experience in the USA (Enoxaparin Sodium, commercialized by Teva Pharmaceutical Industries Ltd., Petah Tikva, Israel, since 2015). Several European authorities more recently approved Chemi Enoxaparin as a biosimilar medicine in 2018, and Italfarmaco subsequently commercialized it in several countries of the EU under different brand names: marketed as Ghemaxan in Belgium, Denmark, Finland, Ireland, Italy, the Netherlands, and Norway; Hepaxane in Germany and Spain; and Havetra in Greece. 

The present study reports the design and main results of the pivotal study used to obtain approval from the European authorities for the commercialization of Chemi Enoxaparin. This pivotal, crossover study compared the bioavailability of enoxaparin from the test investigational medicinal product (IMP) (Chemi Enoxaparin) with the reference IMP (Clexane, manufactured by Sanofi, Maison Alfort, France) when administered as an 80-mg/0.8-mL subcutaneous (s.c.) injection under fasting conditions in healthy subjects. As the determination of pharmacokinetic metrics in LMWH studies present great difficulties, the absorption and elimination of the investigational products in this study are analyzed using pharmacodynamic markers, following the recommendations of the guidelines [8]. 

## Materials and methods 

### Study design 

This study was an open-label, randomized, single-dose, two-period crossover study. The study comprised a screening visit, which was performed within 14 days before the administration of the first dose, and two treatment periods. The two treatment periods lasted 2 days, from the afternoon of the day before administration of the first dose until 36 hours post dose and were separated by a 7-day wash-out period in males and females of non-childbearing potential. The wash-out period was 28 days in female subjects of childbearing potential to allow administration during the same menstrual cycle phase. The South East Wales Research Ethics Committee (United Kingdom) approved the study protocol. The study was performed following the principles contained in the Declaration of Helsinki, the Association of the British Pharmaceutical Industry Guidelines for Phase I Trials, and the International Conference on Harmonization Tripartite Guideline for Good Clinical Practice. Written informed consent was obtained from the subjects before undergoing any study-specific procedure. 

### Selection of the subjects 

We included healthy male or female subjects aged 18 to 55 years with a body mass index of 18 – 30 kg/m^2^. Females of childbearing potential were included if they used 2 effective contraception methods. The following other inclusion criteria were used: no clinically significant abnormal biochemistry, hematology, coagulation factor, or urine examination values within 14 days of the first dose, negative urinary drugs-of-abuse screen within 14 days of the first dose; negative for human immunodeficiency virus, hepatitis B surface, and hepatitis C virus antibodies; no clinically significant abnormality in 12-lead EKG and vital signs within 14 days of the first dose; and the ability to complete the study. 

Subjects were excluded if they weighed < 45 kg (females) or < 57 kg (males), had hypersensitivity to enoxaparin or LMWH, had a history of any relevant medical or psychiatric disorder, had any clinically significant illness within 4 weeks prior to dosing, had recently used non-steroidal anti-inflammatory drugs and/or aspirin, had recent severe trauma, surgery and/or lumbar puncture, were pregnant or lactating, were vegetarian, had donated more than 500 mL of blood/plasma within the previous 3 months, consumed the equivalent of more than 5 cups of coffee per day, were smokers or had stopped smoking within the previous 3 months, or had participated in a study of a new chemical entity or a marketed drug within the previous 4 months or 3 months, respectively. 

Just prior to the first dose (i.e., at baseline), subjects’ eligibility was confirmed. Included subjects had a negative urinary drugs-of-abuse screen and negative urinary pregnancy test. 

### Study medication 

After eligibility was confirmed, subjects were assigned to receive the sequence of a single-dose of Chemi Enoxaparin, followed by a single dose of Clexane (i.e., the reference drug) or the reverse sequence, using a computer-generated randomization schedule and the wash-out periods described above. Each dose contained 80 mg of enoxaparin sodium (equivalent to 8,000 IU anti-factor Xa (anti-FXa activity)) in 0.8 mL water for injection, which was supplied in pre-filled syringes. It was administered s.c. into the left or right side of the abdomen (alternating sides with treatment period), and subjects remained in a semi-recumbent position while receiving each dose and for 4 hours post dose. Doses were administered between 9:00 and 9:57 AM depending on the subject’s number. Subjects were required to fast for at least 10 hours overnight, and they could break the fast 4 hours after dosing. The medication was administered in an unblinded fashion. However, the laboratory staff responsible for drug concentration analyses were blinded to treatment. 

The 80-mg dose was selected because it is within the range of doses approved for the reference drug [4], and it ensured that the biomarkers of anti-coagulation assessed could be measured with appropriate sensitivity. This dose was also the best compromise between the need for a sufficiently high dose to allow for the adequate assessment of anti-FXa and anti-FIIa activity and the need for a dose that was safe in healthy subjects. In addition, a linear relationship between the dose and absorption of enoxaparin was previously demonstrated over a 20-mg to 80-mg dose range in healthy subjects [9]. 

With the exception of hormonal contraceptives, concomitant medications were not allowed from 7 days (prescription medications from 14 days) prior to the first dose until the post-study follow-up visit. Subjects who took any medication during the course of the study were excluded or dropped from the study at the discretion of the investigator. 

### Study assessments 

During the screening period, information on demographics and medical history was collected, and all tests needed to confirm eligibility, including the activated partial thromboplastin time and the prothrombin time/international normalized ratio, were performed. Selection criteria were confirmed, medication history was recorded, and the following tests were performed 1 day before the first dose of the investigational medicinal product was administered: biochemistry; hematology; urinalysis; activated partial thromboplastin time; the prothrombin time/international normalized ratio; drugs of abuse; urine pregnancy test, which was performed prior to dosing at each treatment period; and determination of FSH, LH, estradiol, and progesterone. 

Blood samples for the assessment of pharmacokinetic/pharmacodynamic parameters were collected at pre-dose and at 0.5, 1, 1.5, 2, 3, 4, 5, 6, 8, 12, 16, 24, and 36 hours post dose. Vital signs were recorded at pre-dose and at 2, 4, 6, and 8 hours post dose. The post-study follow-up visit assessment included medical history, biochemistry, hematology, urinalysis, activated partial thromboplastin time, 12-lead EKG, physical examination, and vital signs. Adverse events and concomitant medications were recorded throughout the entire study period. 

### Drug concentration measurements 

The European guidelines on the development of similar biological medicinal products containing LMWHs state that conventional pharmacokinetic (PK) studies cannot be performed due to the heterogeneity of LMWHs, and pharmacodynamic activities should be compared between the biosimilar and the reference LMWH [8]. Therefore, we assessed the activities of anti-FIIa, anti-FXa, thrombin/FIIa generation, tissue factor pathway inhibitor (TFPI), thrombin activatable fibrinolysis inhibitor (TAFI), and TAFI antigen as surrogate biomarkers of anti-coagulation and enoxaparin concentration. 

Blood pharmacodynamic metrices were analyzed during the study at the timepoints specified above. Blood samples (2 × 10 mL) for determination of plasma anti-FIIa, anti-FXa, thrombin/FIIa generation, TFPI, TAFI, and TAFI antigen activity levels were collected from a forearm vein into a citrate sarstedt monovette at each timepoint and kept frozen until analysis. Plasma samples for the measurement of anti-FIIa, anti-FXa, thrombin/FIIa generation, and TAFI activity were analyzed at Simbec Central Laboratories (Merthyr Tydfil, United Kingdom), and plasma samples for the measurement of TFPI activity and TAFI antigen were analyzed using validated assays at Simbec Bioanalytical Department, according to applicable local standard operating procedures. The validation of the analytical method used for the primary outcomes indicated reproducible accuracy throughout the calibration range (0.032 – 0.331 IU/mL for anti-FXa activity and 0.00 – 0.234 IU/mL for anti-FIIa activity) with a spline smoothed fitting. Good inter-batch and intra-batch precision and accuracy was also established following replicate analysis of spiked quality control samples. 

### Statistical analysis 

Individual plasma anti-FIIa, anti-FXa, thrombin/FIIa generation, TFPI, TAFI, and TAFI antigen activity/concentration-time data were evaluated by treatment. Individual and mean activity/concentration-time data were also plotted by treatment on linear and semi-logarithmic scales. The following PK parameters were derived from plasma anti-FIIa, anti-FXa, thrombin/FIIa generation, and TFPI, activity/concentration-time data using Phoenix WinNonlin 6.3, Princeton, NJ, USA: maximum measured plasma activity/concentration (C_max_); time to C_max_ (t_max_); apparent first-order terminal elimination rate constant (λz); apparent first-order terminal elimination half-life (T_1/2_); area under the plasma activity/concentration-time curve from the time 0 to the last measurable concentration (AUC_0–t_); area under the activity/concentration-time curve from time 0 extrapolated to infinity (AUC_0–inf_); minimum plasma activity/concentration (C_min_); time of C_min_ (t_min_); and the residual area (AUC_%ex_). The ratio of anti-FXa/anti-FIIa activity was calculated for each parameter. It was not possible to derive PK parameters from plasma TAFI activity or TAFI antigen. 

The primary PK endpoints were anti-FIIa, anti-FXa C_max_, and AUC_0–t_. Secondary PK endpoints included the following measurements: anti-FIIa and anti-FXa AUC_0–inf_, t_max_, and λz; thrombin/FIIa generation, TFPI, TAFI activity, and TAFI antigen C_max_, AUC_0–t_, and AUC_0–inf_; anti-FIIa, anti-FXa, thrombin/FIIa generation, TFPI, TAFI activity, and TAFI antigen T_1/2_, C_min_, t_min_, and AUC_%ex_; and thrombin/FIIa generation, TFPI, TAFI activity, and TAFI antigen t_max_, λz. The ratio of anti-FXa/anti-FIIa activity was also calculated for each PK parameter. 

Derived PK endpoints are listed and summarized by treatment. The descriptive statistics presented are number dosed (N), number of observations (n), arithmetic mean, arithmetic standard deviation (SD), coefficient of variation (CV%), minimum, median, maximum, and geometric mean (with the exception of t_max_). The results for t_max_ were reverted back to the nominal time. 

Following logarithmic transformation, C_max_, C_min_ (for thrombin/FIIa generation), AUC_0–t_, and AUC_0–inf_ values were subjected to an analysis of variance (ANOVA), including fixed effects for sequence, period, treatment, and subject nested within a sequence. Point estimates and 95% confidence intervals (CI) were constructed for the contrasts between treatments using the residual mean squared error obtained from the ANOVA. We used the 95% CI instead of 90% CI because we evaluated pharmacodynamic parameters. The point and interval estimates were back-transformed to give estimates of the ratios of the geometric least-squared means (LS mean) and corresponding 95% CI. Estimated geometric means were produced for each treatment. 

Bioequivalence was achieved if the 95% CI for the geometric means of C_max_ and AUC_0–t_ were between 80 and 125%. 

An assessment of t_max_ and t_min_ (for thrombin/FIIa generation) was performed using the Wilcoxon matched pairs test [10]. A 95% non-parametric CI was constructed for the median difference in t_max_ based on the method of Campbell and Gardner [11]. 

Sample size calculation of the current study was based on the results of a pilot study (EudraCT Number: 2014-000276-25) in 12 healthy subjects that aimed to examine the within-subject CV% of PK parameters following replicated administration of s.c. Clexane (80 mg) in two different periods. 

For the primary PK endpoints C_max_ and AUC_0–t_ of anti-FIIa and anti-FXa activity, the maximum within-subject CV% was 11.5% (anti-FIIa AUC_0–t_). Assuming a crossover design, a within-subject CV% of 11.5%, a true test-to-reference geometric mean ratio (GMR) of 0.95, and an α error of 5%, a sample of 10 evaluable subjects provide a statistical power of 90% to show bioequivalence (i.e., GMR and corresponding 90% CI within the bioequivalence limits, 0.80 – 1.25) [12]. 

For the secondary PK endpoints AUC_0–t_ and C_min_ of thrombin/FIIa, the maximum within-subject CV% was 29.3% (thrombin/FIIa generation C_min_). Assuming a within-subject CV% of 29.3%, a true test-to-reference GMR between 0.95 and 1.06 and an α error of 5%, a sample of 38 evaluable subjects provide a statistical power of at least 80% to show bioequivalence [12]. 

Subjects withdrawn or dropped out after randomization could not be replaced. Thus, to have at least 38 subjects evaluable, a total of 47 healthy subjects were enrolled in the study. 

Adverse events were coded using the Medical Dictionary for Regulatory Activities (MedDRA) and summarized using absolute and relative frequencies. 

All analyses were performed using SAS version 9.1.3., Cary, NC, USA. 

## Results 

### Subjects’ disposition and characteristics 

A total of 90 subjects were screened, and 47 subjects were randomized to the sequences of treatment. Three of the randomized subjects were withdrawn; 2 subjects were withdrawn because of 2 serious adverse events that were unrelated to the study medications, and 1 subject was withdrawn at the request of the sponsor due to menstrual irregularities. 47 patients were ultimately included in the safety population, and 44 patients were in the pharmacokinetic population (Supplemental Figure 1). 

All subjects were Caucasian and evenly distributed according to sex (23 males and 24 females), with a mean age of 33.2 (SD: 9.98) years, and a mean BMI of 25.4 (SD: 2.71). 

### Anti-FIIa and anti-FXa activities 

**Figure 1.**
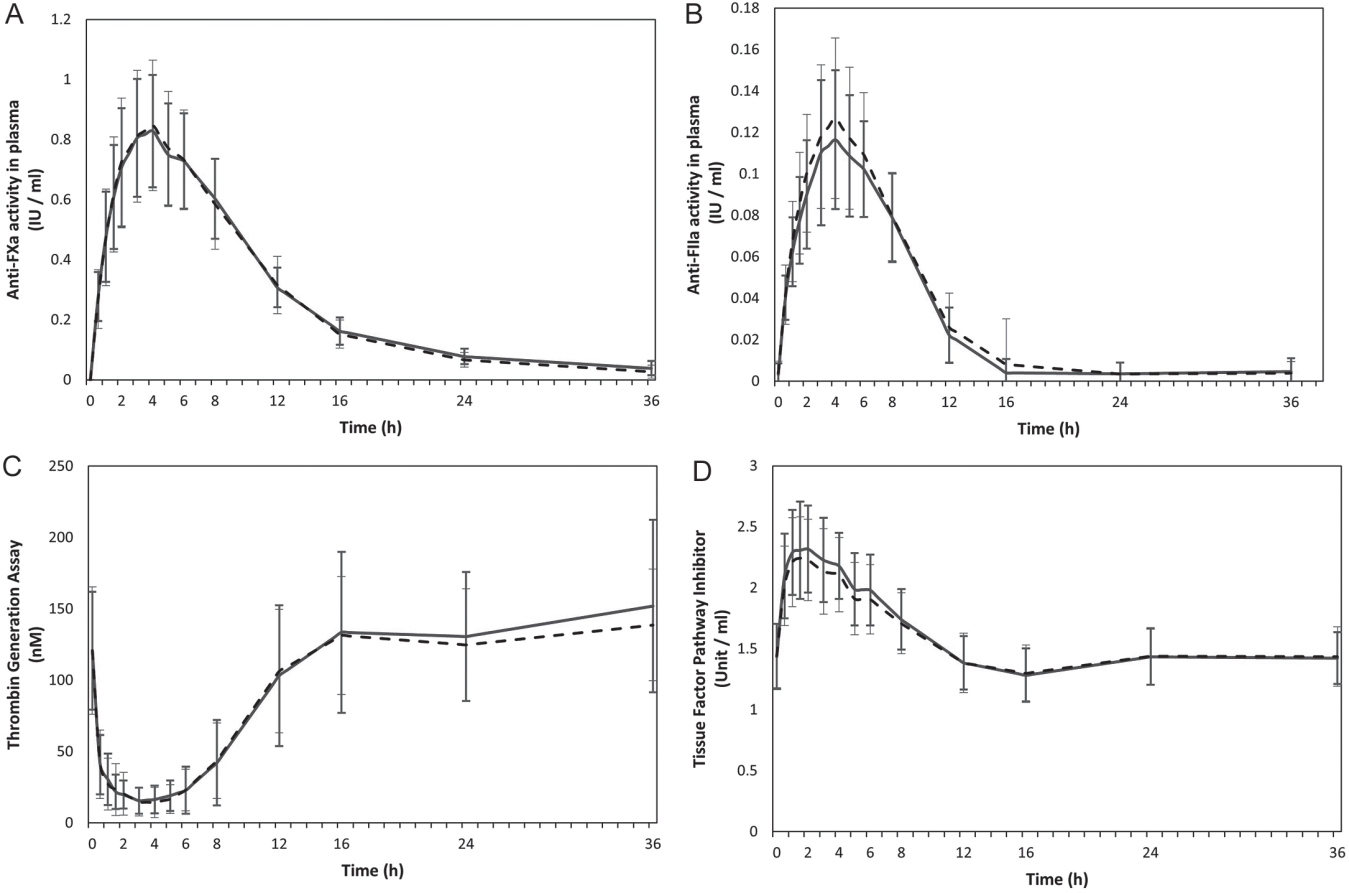
Mean activity-time profiles vs. time curves (linear scale) of single doses of the test or reference drugs after fasting. Mean (SD) plasma anti-FXa (A) and anti-FIIa (B) activity, concentration-time profiles for thrombin generation (C), and activity-time profiles for TFPI levels (D). Test drug is enoxaparin sodium 80 mg (8,000 IU anti-FXa/0.8 mL) manufactured by Chemi SpA (Italfarmaco Group), Milan, Italy (––––). Reference drug is enoxaparin sodium 80 mg (Clexane 8,000 IU anti-FXa/0.8 mL) manufactured by Sanofi, Maison Alfort, France (– – –).

The mean activity-time profiles were similar for both enoxaparin compounds for anti-FXa activity (Figure 1A) (Supplemental Figure 2A) and anti-FIIa activity (Figure 1B) (Supplemental Figure 2B). Regarding the primary endpoints, comparison of the anti-FXa activity of Chemi Enoxaparin with the reference treatment revealed that the 95% CI of the ratios of the geometric LS means were 96.28 – 102.65 IU/mL for C_max_, and 100.67 – 105.15 h×IU/mL for the AUC_0–t_; comparison of the anti-FIIa activity revealed 95% CI of 86.65 – 96.73 IU/mL for the C_max_ and 87.72 – 97.25 h×IU/mL AUC_0–t_ (Table 1). These 95% CIs for the primary endpoints met the criterion for bioequivalence (i.e., 80 – 125%). The inter-individual variability for these parameters was low, with a coefficient of variation ranging from 5.8 to 12.8%. 

Other key secondary endpoints of the anti-FIIa and anti-FXa activity also met the bioequivalence criterion, including the AUC_0–inf_ for the anti-FIIa activity and anti-FXa activity, and the C_max_, AUC_0–t_, and AUC_0–inf_ of the ratio anti-FXa/anti-FIIa activity were not different between Chemi Enoxaparin and the reference drug. The results for t_max_ also met the bioequivalence criterion as for the anti-FIIa and anti-FXa activity and for the ratio anti-FXa/anti-FIIa activity (Table 2). Further secondary outcomes can be found in Supplemental Tables 1 and 2. 

### Thrombin generation assay 

Plasma concentration time data for thrombin/FIIa generation also showed similar profiles for Chemi Enoxaparin with the reference treatment (Figure 1C) (Supplemental Figure 2C). The ratio of the geometric LS means of AUC_0–t_ for thrombin/FIIa generation for the comparison of Chemi Enoxaparin with the reference drug met the criterion for bioequivalence, but the ratio for the C_min_ did not meet the criterion, with a 95% CI of 102.29 – 129.08 nM (Table 2). 

### Tissue factor pathway inhibitor activity 

The mean activity-time profiles for TFPI activity were also similar for Chemi Enoxaparin and the reference enoxaparin (Figure 1D) (Supplementary Figure 2D), and all derived PK parameters met the bioequivalence criterion, with no difference between the compounds in the t_max_ (Table 2). TFPI activity values were not corrected for baseline since we found substantial increases over baseline endogenous levels. 

### Thrombin activatable fibrinolysis inhibitor activity and thrombin activatable fibrinolysis inhibitor antigen 

There was little difference in TAFI activity and TAFI antigen between Chemi Enoxaparin (test IMP) and the reference IMP, as reflected by the similar activity-time profiles (Supplemental Figure 3). There was little change in TAFI activity and TAFI antigen overall during the 36-hour sampling period. 

### Safety and tolerability 

The number of subjects reporting at least 1 treatment emergent adverse event (TEAE) was low (i.e., 15 TEAEs reported by 13 subjects) and similar for both compounds. All TEAEs were considered unrelated to the study medication, and most (i.e., 10 of the 15 TEAEs) were considered to be of mild severity. Two subjects reported 2 serious adverse events during the study: fibrous dysplasia after the reference drug administration and cellulitis following Chemi Enoxaparin administration. However, these events were not considered related with the study medication. The subject with cellulitis recovered, and the subject with fibrous dysplasia was awaiting surgical intervention at the time the clinical study report was closed. 

There were no clinically significant biochemistry, hematology, or urinalysis values reported during the study. There was little difference in mean activated partial thromboplastin time (APTT) values following the administration of Chemi Enoxaparin and the reference treatment, which was reflected in the similar values on day –1 and 36 hours post dose of each treatment period. Therefore, the mean change from baseline to 36 hours post dose in the APTT was –1.10 seconds with Chemi Enoxaparin and –1.31 seconds with the reference IMP. Physical examinations and vital signs revealed that 1 subject had a clinically significant physical examination finding of eczema, which was reported as a TEAE that was mild in severity and considered unrelated to the study medication. There were no other clinically significant findings on physical examination or the 12-lead EKG values reported during the study. 

## Discussion 

This randomized, crossover, open-label study showed that Chemi Enoxaparin is equivalent to the reference drug at a dose of 80 mg s.c. based on the results of the primary PK endpoints anti-FIIa and anti-FXa activity C_max_ and AUC_0–t_. Bioequivalence was also shown for all secondary PK endpoints, except thrombin/FIIa generation C_min_. Our results indicated a rapid and similar absorption of Chemi Enoxaparin compared to the reference drug. Both compounds were well tolerated without safety issues. 

Anti-FIIa and anti-FXa activity are the key assessments for establishing bioequivalence because these anti-thrombin assays are highly specific for heparins. These potency assays are recommended by the European and U.S. Pharmacopeias [1], and they are required assessments according to the European Guidelines for the development of similar LMWH [8]. Chemi enoxaparin compared to the reference treatment at an 80-mg s.c. dose, showing a geometric LS mean 95% CI that fell within 80 – 125% for the primary PK endpoints of anti-FIIa and anti-FXa activity C_max_ and AUC_0–t_. European guidelines also require the comparison of the ratios of anti-FXa/anti-FIIa activity and TFPI activity [8]. Our results demonstrated bioequivalence for anti-FIIa and anti-FXa activity (AUC_0–inf_) and the ratio of anti-FXa/anti-FIIa activity (C_max_, AUC_0–t_, and AUC_0–inf_). TFPI binds to FXa and inactivates the tissue factor-FVIIa-FXa complex, and each LMWH has a distinct TFPI profile [13]. We showed the bioequivalence of Chemi Enoxaparin for TFPI activity (C_max_, AUC_0–t_, and AUC_0–inf_). Although similar, thrombin/FIIa generation C_min_ for Chemi Enoxaparin (11.798 nM) was not considered bioequivalent to the reference drug (10.267 nM), with an upper CI that was just outside the 125% limit (the 95% CI was 102.29 – 129.08 nM). However, the AUC_0–t_ was considered bioequivalent to the reference drug (95% CI: 97.51 – 108.28). Notably, thrombin/FIIa generation C_min_ showed the greatest inter-individual variation, with a CV of 27.5%, which may partially explain this result. There was no difference in the t_max_ for anti-FIIa activity, anti-FXa activity, the ratio of anti-FXa/anti-FIIa activity, and TFPI activity or t_min_ for thrombin/FIIa generation between Chemi Enoxaparin and the reference drug, which indicates no difference in the rate of absorption between the two formulations. 

Subcutaneous Chemi Enoxaparin (80 mg) was safe and well-tolerated, with a similar TEAE, biochemistry, hematology, coagulation (APTT), and vital signs profile to the reference drug. 

Previous LMWH bioequivalence trials have been published for biosimilar medicines authorized by the European authorities [14, 15]. However, these have either failed to meet the bioequivalence criteria for TFPI [14] or have published only the data related to the regulatory requirements (anti-FXa, anti-FIIa, and TFPI) [15]. Our study not only shows how Chemi Enoxaparin (80 mg) meets the bioequivalence criteria for all the required outcomes (anti-FXa activity, anti-FIIa activity, the ratio of anti-FXa/anti-FIIa activities, and TFPI activity), but also presents additional results involving these outcomes (such as λz, T_1/2_, t_max_, and t_min_) and other outcomes (thrombin/FIIa generation) that may further help to comprehend the comparability of this medicinal product and the reference product. 

We used an open-label design for this trial because the primary and secondary endpoints were pharmacodynamic parameters that are not susceptible to the influence of the investigators’ or subjects’ perceptions. The personnel in charge of the pharmacodynamic determinations were blinded to the study medications. Therefore, it is highly unlikely that this design introduced an evaluation bias. This design is frequently used in trials demonstrating the bioequivalence of biosimilars of LMWH [16, 17] and other biologics [18, 19]. 

Our results show that Chemi Enoxaparin was safe and well tolerated, with a similar profile to the reference product. Although the information on safety and tolerability from healthy subjects has limitations, our results are further supported by the quite lengthy clinical experience with Chemi Enoxaparin on the market. Chemi enoxaparin has a longer experience in actual clinical practice compared to other enoxaparin biosimilars marketed in Europe, especially due to its previous commercialization in the U.S., and no unexpected events and/or safety profile differences from the reference product were shown. 

## Conclusion 

As for the regulatory implications of these data, they have been used in several European countries as pivotal clinical evidence for requesting marketing authorization of the biosimilar enoxaparin manufactured by Chemi. 

In conclusion, this single-dose study demonstrated that Chemi Enoxaparin (80 mg, s.c.) is bioequivalent to the branded Clexane (80 mg, s.c.), and both compounds show similar tolerability and safety profiles. 

## Acknowledgment 

The authors would like to thank Fernando Rico-Villademoros (Cociente SL, Madrid, Spain) for providing medical writing assistance, to ITF Research Pharma S.L.U for providing funding, and to Eva García Aguilar (ITF Research Pharma S.L.U) for her editorial coordination. 

## Data availability 

Due to the nature of this research, participants of this study did not agree for their data to be shared publicly, so supporting data is not available. 

## Authors’ contributions 

S.F. and P.B. contributed to the design and implementation of the research, to the analysis of the results and reviewed and approved the manuscript. J.L.M.B. and C.N.M. contributed to the analysis of the results and to the writing of the manuscript. 

## Funding 

This work was supported by ITF Research Pharma S.L.U. (Italfarmaco) and Chemi S.p.A. (Italfarmaco). 

## Conflict of interest 

SF reports no conflict of interest. JLMB and CNM are full-time employees at ITF Research Pharma S.L.U. PB is a full-time employee at Italfarmaco S.p.A. 

**Table 1.** Primary pharmacodynamic endpoints.

Endpoint	Chemi Enoxaparin (80 g/0.8 mL) N = 44	Reference drug (80 g/0.8 mL) N = 44	Test vs. reference	CV% from ANOVA
Anti-FXa activity				
	*Geometric LS mean*	*Geometric LS mean*	*Geometric LS mean ratio (95% CI)*	
C_max_ (IU/mL)	0.840	0.845	99.42 (96.28 – 102.65)	7.5
AUC_0–t_ (h×IU/mL)	9.484	9.218	102.89 (100.67 – 105.15)	5.1
AUC_0–Inf_ (h×IU/mL)	10.142	9.728	104.26 (101.68 – 106.90)	5.8
	*Median*	*Median*	*Median difference * *(95% CI) [p-value]*	
t_max_ (h)	4.0	4.0	0.0 (–0.50 – 0.50) [0.7642]	na
Anti-FIIa activity				
	*Geometric LS mean*	*Geometric LS mean*	*Geometric LS mean ratio (95% CI)*	
C_max_ (IU/mL)	0.114	0.125	91.55 (86.65 – 96.73)	12.8
AUC_0–t_ (h×IU/mL)	0.984	1.066	92.37 (87.72 – 97.25)	12.0
AUC_0–Inf_ (h×IU/mL)	1.114	1.232	90.44 (85.38 – 95.80)	11.8
	*Median*	*Median*	*Median difference * *(95% CI) [p-value]*	
t_max_ (h)	4.0	4.0	0.0 (–0.50 – 0.50) [0.9464]	na

ANOVA = analysis of variance; CI = confidence interval; CV = coefficient of variation; LS = least square; na = not applicable.

**Table 2.** Secondary pharmacodynamic parameters.

Endpoint	Chemi Enoxaparin (80 g/0.8 mL) N = 44	Reference drug (80 g/0.8 mL) N = 44	Test vs. reference	CV% from ANOVA
Ratio anti-FXa/anti-FIIa activity				
	*Geometric LS mean*	*Geometric LS mean*	*Geometric LS mean ratio (95% CI)*	
C_max_ (IU/mL)	7.368	6.785	108.59 (103.93 – 113.46)	10.2
AUC_0–t_ (h×IU/mL)	9.634	8.649	111.39 (105.89 – 117.17)	11.8
AUC_0–inf_ (h×IU/mL)	9.110	8.005	113.82 (107.47 – 120.53)	11.7
	*Median*	*Median*	*Median difference * *(95% CI) [p-value]*	
t_max_ (h)	1.0	1.0	0.0 (–0.13 – 0.13) [0.9217]	na
Tissue factor pathway inhibitor (TFPI) activity				
	*Geometric LS mean*	*Geometric LS mean*	*Geometric LS mean ratio (95% CI)*	
C_max_ (Unit/mL)	2.408	2.317	103.94 (101.22 – 106.73)	6.2
AUC_0–t_ (h×Unit/mL)	55.645	55.172	100.86 (99.27 – 102.47)	3.7
AUC_0–inf_ (h×Unit/mL)	143.434	150.772	95.13 (85.70 – 105.60)	9.7
	*Median*	*Median*	*Median difference * *(95% CI) [p-value]*	
t_max_ (h)	1.5	1.5	0.0 (–0.25 – 0.50) [0.6857]	na
Thrombin/FIIa generation				
	*Geometric LS mean*	*Geometric LS mean*	*Geometric LS mean ratio (95% CI)*	
C_min_ (nM)	11.798	10.267	114.91 (102.29 – 129.08)	27.5
AUC_0–t_ (h×nM)	3,551.186	3,455.956	102.76 (97.51 – 108.28)	12.2
	*Median*	*Median*	*Median difference * *(95% CI) [p-value]*	
t_min_ (h)	4.0	4.0	0.0 (–0.50 – 0.50) [0.9464]	na

ANOVA = analysis of variance; CI = confidence interval; CV = coefficient of variation; LS = least square; na = not applicable. Note: It was not possible to derive PD parameters for TAFI activity or TAFI antigen from the concentration-time profiles.

## Supplemental material

Supplemental material Supplemental Tables and Figures
