# Supplementary material for: Bioavailability study of Enoxaparin Sodium Chemi (80 mg/0.8 mL) and Clexane (80 mg/0.8 mL) subcutaneous injection in healthy adults
Source: Int J Clin Pharmacol Ther. 2021 Aug 17;59(11):734–44. doi: 10.5414/CP204022 (PMC8554289; doi:10.5414/CP204022)
Supplement: Supplemental material [file intjclinpharmacol-59-734-S01.pdf]

Bioavailability study of Enoxaparin Sodium Chemi (80 mg/0.8 mL) and Clexane® (80 mg/0.8 mL) subcutaneous injection in healthy adults

Supplementary information

Treatment	Anti-FXa Activity									
	Summary Statistic	**C_{max}** (IU/mL)	**T_{max}** (h)	**λz** (1/h)	**$t_{1/2}$** (h)	**AUC_{0-t}** (h*IU/mL)	**AUC_{0-Inf}** (h*IU/mL)	**$AUC_{\%ex}$** (%)	**C_{min}** (IU/mL)	**T_{min}** (h)
**Test IMP: Chemi Enoxaparin** (80 mg /0.8 mL) (n=44)	*Mean*	0.859	4.023	0.096	8.476	9.626	10.279	6.452	0	0
	SD	0.181	0.952	0.043	3.227	1.686	1.714	2.681	0	0
	Min	0.522	2	0.042	3.085	6.986	7.777	2.845	0	0
	Median	0.873	4	0.085	8.140	9.603	10.197	5.809	0	0
	Max	1.236	6	0.225	16.447	13.402	14.407	13.777	0	0
	CV%	21.0	23.7	44.2	38.1	17.5	16.7	41.6	NC	NC
	Geo. Mean	0.840	N/a	0.088	7.849	9.484	10.142	5.949	NC	NC
**Reference IMP: Clexane** (80 mg /0.8 mL) (n=44)	*Mean*	0.870	4.0	0.113	6.957	9.393	9.920	4.968	0	0
	SD	0.209	0.715	0.043	2.426	1.839	1.861	1.809	0	0
	Min	0.551	3	0.053	2.873	6.212	6.704	2.089	0	0
	Median	0.871	4	0.096	7.225	9.189	9.641	4.779	0	0
	Max	1.312	6	0.241	13.199	13.498	13.786	8.583	0	0
	CV%	24.1	17.9	38.3	34.9	19.6	18.8	36.4	NC	NC
	Geo. Mean	0.845	N/a	0.106	6.532	9.218	9.749	4.640	NC	NC

N/a = not applicable, NC = not calculated.

Supplementary Table 1. Anti-FXa activity pharmacokinetic determinations.

Treatment	Anti-FIIa Activity									
	Summary Statistic	**C_{max}** (IU/mL)	**T_{max}** (h)	**λz** (1/h)	**$t_{1/2}$** (h)	**AUC_{0-t}** (h*IU/mL)	**AUC_{0-Inf}** (h*IU/mL)	**$AUC_{\%ex}$** (%)	**C_{min}** (IU/mL)	**T_{min}** (h)
**Test IMP: Chemi Enoxaparin** (80 mg /0.8 mL) (n=44)	*Mean*	0.119	4.182	0.211	4.621	1.023	1.149	12.059	0.002	4.180
	SD	0.035	0.971	0.104	2.980	0.288	0.292	7.688	0.004	8.538
	Min	0.059	2	0.070	1.877	0.537	0.690	3.342	0	0
	Median	0.116	4	0.225	3.081	0.995	1.131	11.392	0	0
	Max	0.227	6	0.369	9.875	1.639	1.781	36.516	0.010	24
	CV%	29.1	23.2	49.4	64.5	28.2	25.5	63.8	233.6	204.2
	Geo. Mean	0.114	N/a	0.181	3.834	0.984	1.113	9.968	0.012	20.04
**Reference IMP: Clexane** (80 mg /0.8 mL) (n=44)	*Mean*	0.130	4.364	0.194	6.505	1.110	1.293	12.091	0.002	6.910
	SD	0.037	1.954	0.116	8.675	0.317	0.404	9.029	0.004	12.211
	Min	0.073	3	0.013	1.584	0.618	0.747	1.966	0	0
	Median	0.125	4	0.179	3.863	1.020	1.282	9.679	0	0
	Max	0.223	16	0.437	53.976	1.784	2.595	39.013	0.010	36
	CV%	28.7	44.8	59.9	133.4	28.5	31.3	74.7	234.3	176.7
	Geo. Mean	0.125	N/a	0.154	4.5	1.066	1.239	9.441	0.011	24

N/a = not applicable.

Supplementary Table 2. Anti-FIIa activity pharmacokinetic determinations.

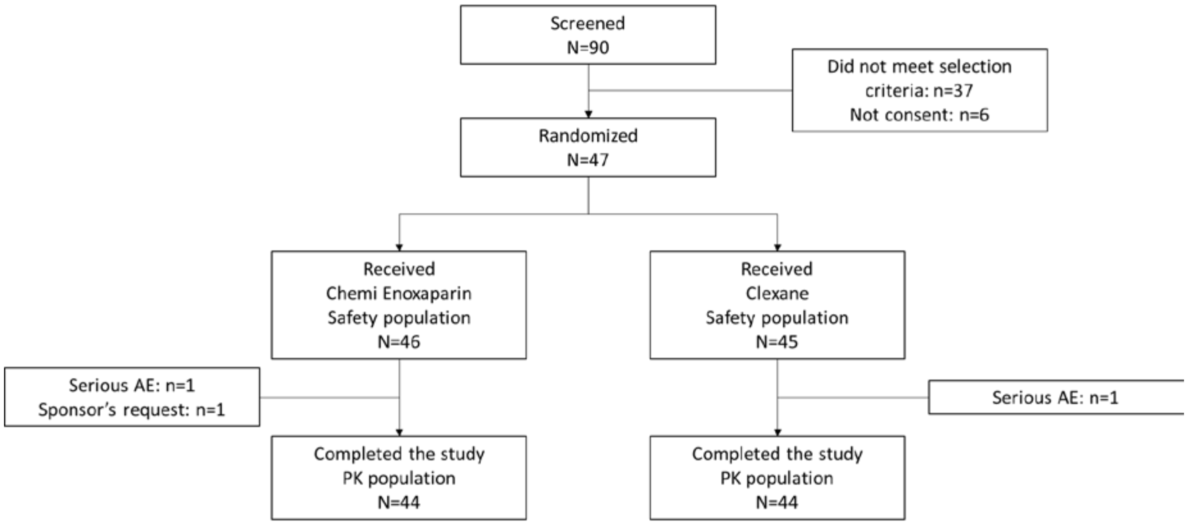

Supplementary Figure 1. Patient disposition.

SF2A Mean Activity-Time Profiles for Anti-FXa Activity

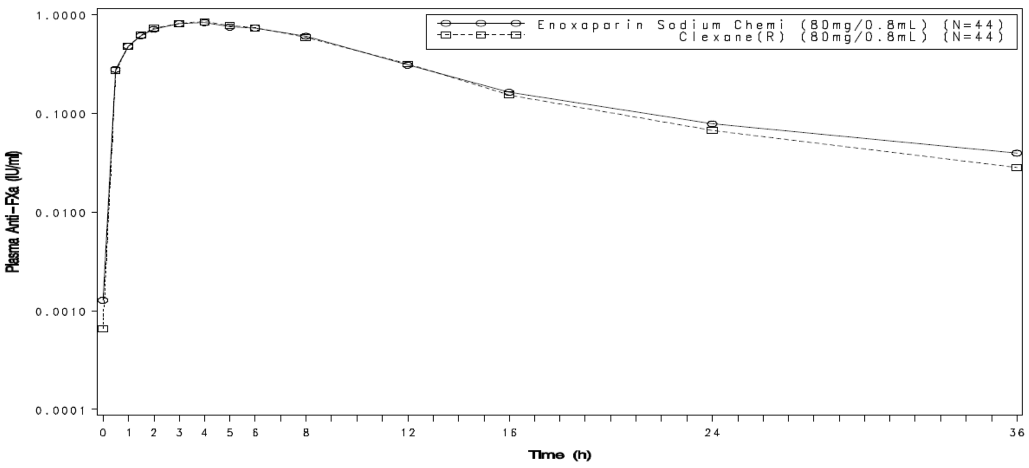

SF2B Mean Activity-Time Profiles for Anti-FIIa Activity

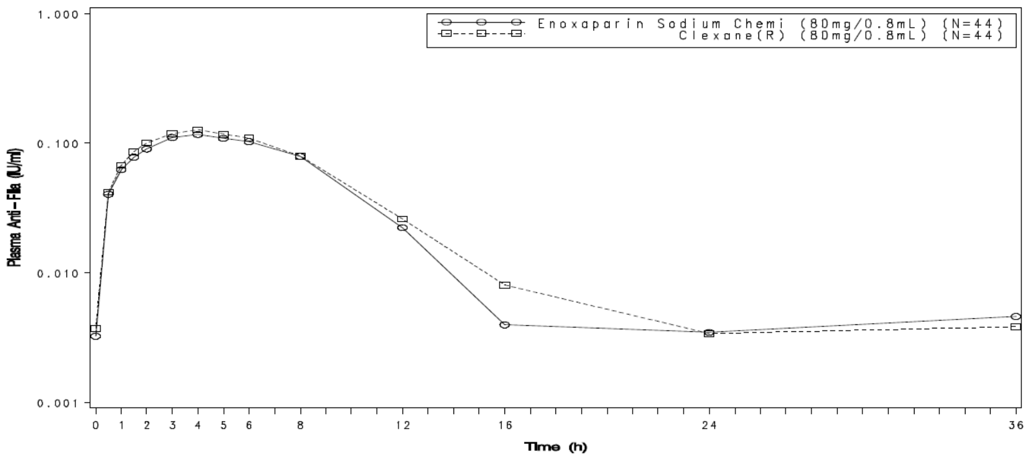

SF2C Mean Concentration-Time Profiles for Thrombin/FIIa Generation

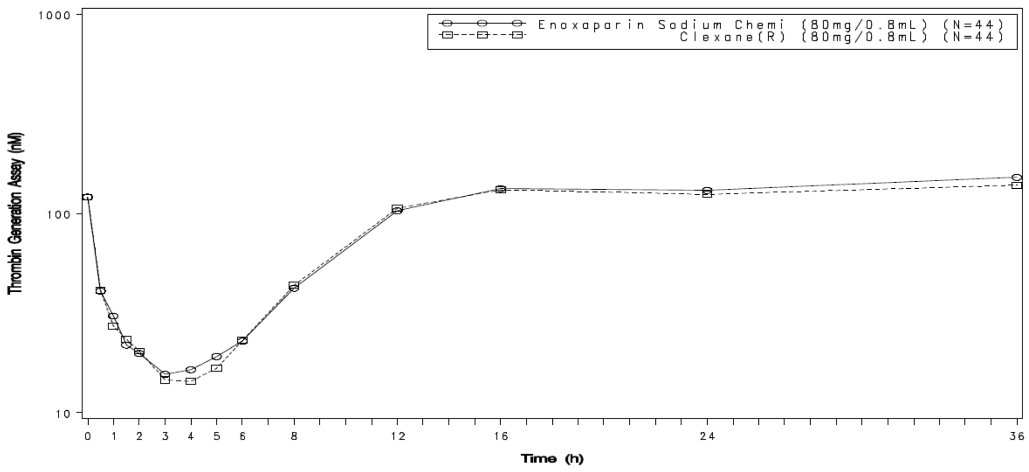

SF2D Mean Activity-Time Profiles for TFPI Activity

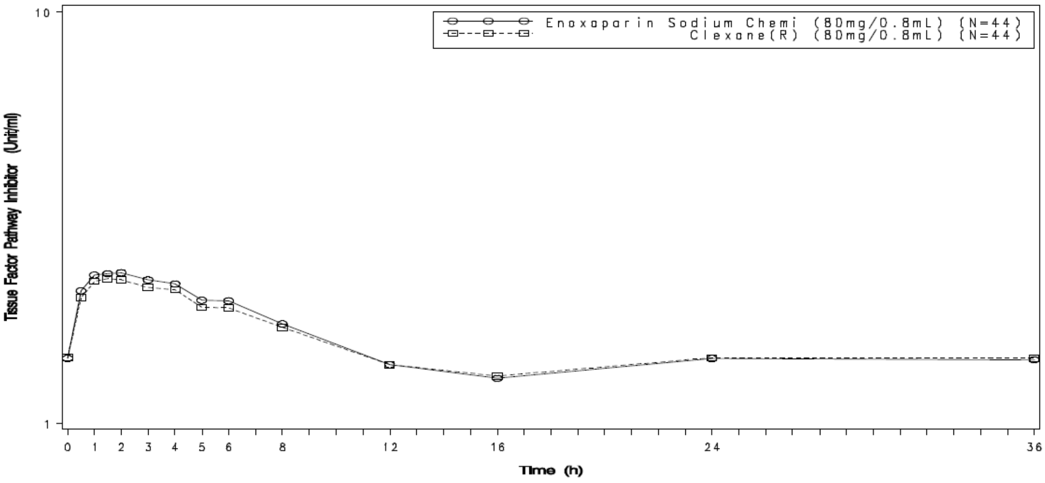

Supplementary Figure 2. Mean activity-time profiles vs. time curves (semilogarithmic scale) after fasting of single doses of the test or reference drugs.

SF3A Mean (SD) thrombin activatable fibrinolysis inhibitor activity

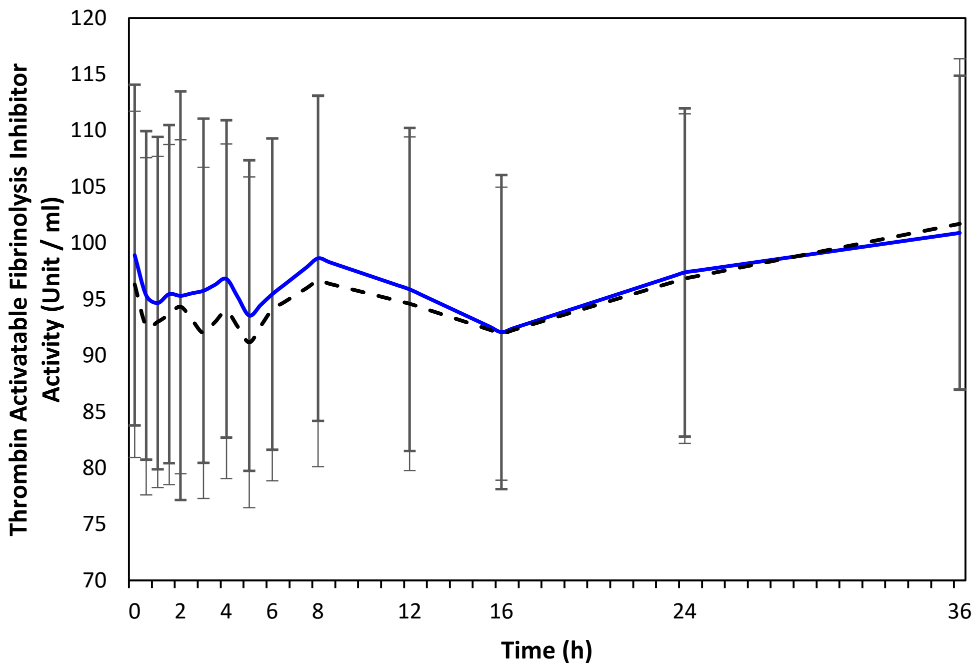

SF3B Mean (SD) thrombin activatable fibrinolysis inhibitor antigen

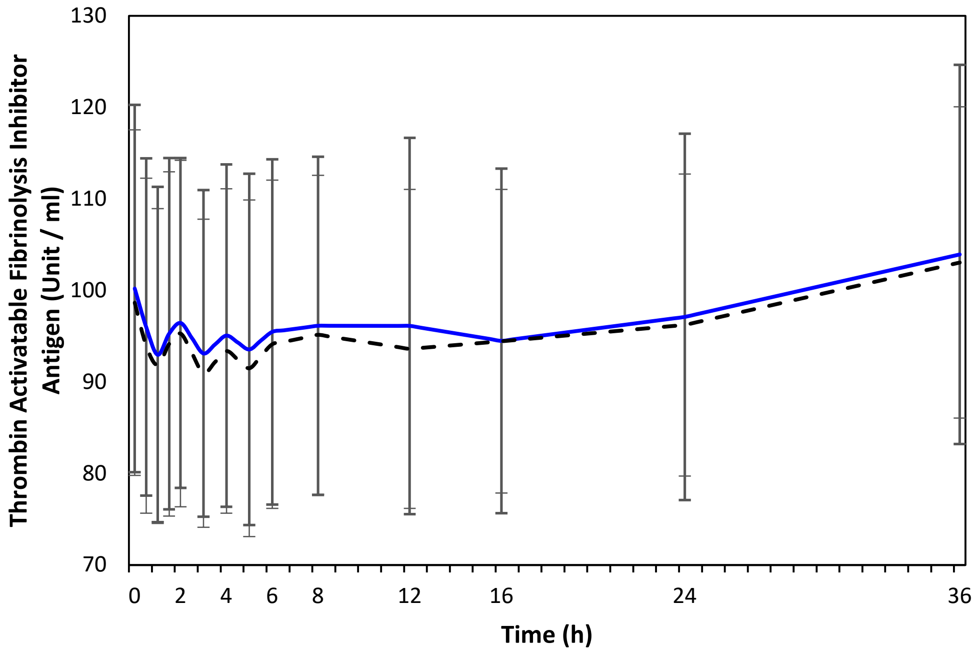

Supplementary Figure 3. Mean activity-time profiles vs. time curves (linear scale) after fasting of single doses of the test or reference drugs. Mean (SD) TAFI activity (A) and TAFI antigen (B).

Test drug is enoxaparin sodium 80 mg (8.000 IU anti-FXa/0.8 ml) manufactured by Chemi SpA (Italfarmaco Group), Milan, Italy (——). Reference drug is enoxaparin sodium 80 mg (Clexane® 8.000 IU anti-FXa/0.8 ml) manufactured by Sanofi, Maison Alfort, France (- - -).
